# A Textbook of Bacteriology

**Published:** 1911-03

**Authors:** 


### BOOKS AND AUTHORS

---

**A Textbook of Bacteriology.** By **Philip Hanson Hiss,** Jr., M.D., Professor of Bacteriology, College of Physicians and Surgeons, Columbia University, New York, and **Hans Zinsser**, M.D., Associate Professor in Charge of Bacteriology, Leland Stanford, Jr., University, Palo Alto, Cal. Octavo, pp. 745. Illustrated. New York and London: D. Appleton & Co. 1910. (Cloth, $3.75.)

This book shows the width of the sympathy of workers in science,—the Atlantic and the Pacific, the east and the west, New York and California, Hiss and Zinsser, co-workers, authors. And the work is worthy its wide foundation.

The usual subjects of textbooks of this class are fully treated and copiously illustrated. An entire section of five chapters is devoted to the consideration of diseases of unknown etiology. We believe the authors will take advantage of future editions to strengthen an important chapter—Section II, Chapter XI., "Infection and Immunity, fundamental factors of pathogenicity and infection"—by laying greater emphasis upon the role of the chemistry of pathology, and by giving their readers and students fuller opportunites to study the more recent literature as to the role of catalysts in general and with greater particularity the pathogenic catalysts.

It seems singular that Abderhalden's name does not appear in the index of authors and that the many pertinent facts brought out in this chapter on ferments are not given extended consideration. When Abderhalden says: "The chemical decompositions in the tissues do not take place beside the 'physiological processes' but are bound up with them," * * * "We have in mind here the great field of infectious diseases and the changes in the metabolism of the cells which they occasion," * * * "Pathology, also, in a broad sense, seeks to unite itself more and more with the field of physiological chemistry," * * * "It becomes more and more evident that pathological changes in the tissues and cells should not be judged entirely from a morphological standpoint," he utters words of wisdom, words well worthy the attention of writers of works on bacteriology of disease, of pathogenic bacteria.

The author's discussion on anaphylaxis is the best we remember to have seen and this subject is now enriched or beclouded, as the terms are rightly or wrongly used, with several new words. —namely, antianaphylaxis, anaphylactic, aphylactic, anaphylactin, sensibilisinogen, sensibilisin, antisensibilisin. These may look and even sound formidable. Hiss and Zinsser, authors, use them to throw valuable light upon a very important factor in the production of disease,—anaphylaxis. H. R. H.

